# Phthalates and phthalate metabolites in urine from Tianjin and implications for platelet mitochondrial DNA methylation

**DOI:** 10.3389/fpubh.2023.1108555

**Published:** 2023-04-26

**Authors:** Weixia Li, Liqiong Guo, Junkai Fang, Lei Zhao, Shanjun Song, Tao Fang, Chenguang Li, Lei Wang, Penghui Li

**Affiliations:** ^1^School of Environmental Science and Safety Engineering, Tianjin University of Technology, Tianjin, China; ^2^Tianjin Fourth Central Hospital, Tianjin, China; ^3^Tianjin Key Laboratory of Hazardous Waste Safety Disposal and Recycling Technology, Tianjin, China; ^4^Institute of Disaster and Emergency Medicine, Tianjin University, Tianjin, China; ^5^Tianjin Key Laboratory of Disaster Medicine Technology, Tianjin University, Tianjin, China; ^6^Wenzhou Safety (Emergency) Institute, Tianjin University, Wenzhou, China; ^7^Tianjin Institute of Medical and Pharmaceutical Sciences, Tianjin, China; ^8^National Institute of Metrology, Beijing, China; ^9^Hebei Research Center for Geoanalysis, Baoding, Hebei, China

**Keywords:** phthalate, phthalate metabolites, health risk assessment, *mtDNA* methylation, cardiovascular diseases

## Abstract

**Background:**

Phthalates (PAEs) are important synthetic substances in plastics, attracting much attention due to their potential effects on the cardiovascular system.

**Methods:**

In this study, urine and blood samples from 39 individuals were collected in Tianjin, China. Phthalates and phthalate metabolites (mPAEs) were analyzed using gas chromatography-mass spectrometry (GC-MS) and high-performance liquid chromatography-mass spectrometry (HPLC-MS), respectively. The polymerase chain reaction (PCR) products from bisulfite-treated mitochondrial DNA (*mtDNA*) samples were analyzed using pyrosequencing technology.

**Results:**

The detection frequencies for 9 PAEs varied from 2.56 to 92.31%, and those for 10 mPAEs varied from 30.77 to 100%. The estimated daily intakes (EDIs) and cumulative risk of PAEs were calculated based on the experimental statistics of urinary PAEs and mPAEs. For PAEs, the HI_RfD_ (hazard index corresponding to reference doses) values of 10.26% of participants and the HI_TDI_ (hazard index corresponding to tolerable daily intake) values of 30.77% of participants were estimated to exceed 1, suggesting a relatively high exposure risk. The *mtDNA* methylation levels in the *MT-ATP8* and *MT-ND5* were observed to be lower than in the *MT-ATP6*. Mono-ethyl phthalate (MEP) and *MT-ATP8* were positively correlated with triglyceride levels (*p* < 0.05). Based on the association of PAEs, *mtDNA* methylation, and triglycerides, the mediating role of *mtDNA* methylation between PAEs and cardiovascular diseases (CVDs) was analyzed in this study, but no mediated effect was observed.

**Conclusion:**

The effects of PAE exposure on cardiovascular diseases (CVDs) should be investigated further.

## 1. Introduction

Phthalates (PAEs) are synthetic organic compounds that are widely used as additives in plastic products, such as food packing, personal care products, and medical devices (1). Since PAEs are not chemically bound to plastics, they are easily released into the environment (2). Therefore, PAEs have been widely detected in environmental matrices, such as soil, sediment, food, and air (3), and humans can be exposed to PAEs through various routes, including ingestion, inhalation, and dermal absorption from the environment (4, 5). Urine has been recognized as a suitable indicating matrix for exposure estimation due to rapid metabolization and short half-lives (< 24 h) of PAEs in it (6, 7). Most recent studies on estimated daily intakes (EDIs) of PAEs focused on mPAEs (1, 5, 8), but one study detected the existence of PAEs in urine (9). Thus, the EDIs of PAEs may be underestimated.

The toxic effects of PAEs are causing increasing public concern, with several epidemiological studies revealing that PAEs can lead to oxidative stress (10–13) and cardiovascular diseases (CVDs) (14–17). Virani et al. reported that high levels of triglycerides, total cholesterol (TC), low-density lipoprotein cholesterol (LDL-C), and lower levels of high-density lipoprotein cholesterol (HDL-C) are well-known CVD risk factors (18). In addition, Mínguez-Alarcón et al. found that certain PAE exposures were associated with lipid biomarkers (HDL-C, triglycerides, LDL-C, and TC) (19). However, studies about the relationships between PAE exposure and CVD were limited, and the potential biological mechanism remains under-recognized.

Increasing studies have confirmed that epigenetic patterns can be modified by some environmental exposures, and DNA methylation is the most studied and best-understood type of epigenetic modification (20, 21). A study has shown that DNA methylation is associated with benzene, dichlorodiphenyl trichloroethane, and polychlorinated biphenyls (22). Tian et al. (23) found that sperm LINE-1 DNA methylation levels were negatively correlated with bis(2-ethylhexyl) phthalate (DEHP) exposure, mediating the relationship between DEHP and sperm motility.

Over the years, methylation in mitochondrial DNA (*mtDNA*) has drawn attention. As *mtDNA* lacks a protector protein and effective repair mechanism, it is more vulnerable to reactive oxygen species (ROS) and mutations than nuclear DNA (24–26). An ENVIRONAGE birth cohort study suggested that *mtDNA* reacts to particulate matter that leads to ROS (27). *mtDNA* methylation could affect the normal function of mitochondria, causing mitochondrial damage and dysfunction, and has been recognized as a biomarker in many human diseases. Han et al. showed that *mtDNA* methylation may be a potential biomarker for predicting breast cancer risk (28). Stoccoro et al. (29) found *mtDNA* methylation differences in obese individuals and patients with colorectal cancer and CVD (30). Therefore, *mtDNA* methylation, as an epigenetic pattern, can be considered a potential mechanism of environmental exposures and CVDs.

Despite emerging interests and breakthroughs in *mtDNA* methylation in recent years, environmental exposures influencing CVDs through epigenetic mechanisms have yet to be thoroughly explored, and investigations on humans are still urgently needed. In this study, urine and blood samples from 39 participants were collected in Tianjin. The main objectives of this study are (1) the determination of PAEs and mPAEs in urine samples, (2) the investigation of the relationships of PAEs and mPAEs with lipid levels and *mtDNA* methylations, and (3) the assessment of the EDIs and potential health risks.

## 2. Materials and methods

### 2.1. Study population

In this study, we recruited 39 men (aged 27–43 years, referred by a physician) from Project ELEFANT (26, 31–35). At recruitment, all participants excluded autoimmune diseases, cardiovascular diseases, severe liver diseases, thyroid diseases, and blood diseases, and completed a physical examination under the guidance of nurses, signed an informed consent form, and filled in basic information (including age and BMI) listed in Table 1. This project complies with the Human Medical Ethic Review Law of the Ministry of Health and the Declaration of Helsinki. All procedures and study protocols were authorized by the ethics committee of Tianjin Medical University.

**Table 1:** Characteristics of the subjects.

**Characteristics**	**Mean (Min-Max) or *n* (%)**
**Gender**	
Man	39 (100%)
Age (years)	27–43 (34.7)
**Body mass index (BMI) (kg/m** ^2^ **)**	
< 18.5	2 (5.1%)
18.5–23.9	12 (30.8%)
>23.9	25 (64.1)
**Years of service (years)**	
≤2	7 (17.9%)
>2	32 (82.1%)

### 2.2. Measurement of urinary PAEs and mPAEs

In this study, we monitored 9 PAEs and 10 mPAEs, including di-methyl phthalate (DMP), di-ethyl phthalate (DEP), di-iso-butyl phthalate (DiBP), di-butyl phthalate (DBP), 1,2-benzenedicarboxylicacid (BMPP), di-pentyl phthalate (DPP), diethylhexyl phthalate (DEHP), di-n-octyl phthalate (DNOP), di-phenyl phthalate (DPHP), mono-methyl phthalate (MMP), mono-ethyl phthalate (MEP), mono-isobutyl phthalate (MiBP), mono-n-butyl phthalate (MBP), mono-ethyl phthalate (MEHP), mono-benzyl phthalate (MBzP), mono-octyl phthalate (MOP), mono-(2-ethyl-5-hydroxy-hexyl) phthalate (MEHHP), mono-(2-ethyl-5-oxo-hexyl) phthalate (MEOHP), and mono-(2-ethyl-5-carboxy-pentyl) phthalate (MECPP). To avoid the transfer of PAEs from PVC into urine samples, all materials were kept away from plastic vessels. Urine samples were collected, stored, and analyzed using glass tubes. Urine samples were gathered and stored at −20°C until analysis. To determine PAEs, 10 μl of internal standard (D4-DEHP, D4-DOP, and D4-DBP mixed solution, 10 μg/ml, Sigma) and 0.5 ml n-hexane was added to 0.5 ml of urine sample, and the sample mix solution was vortexed for 10 min. The sample mixture solution was centrifuged at 9,000 r/min for 10 min. The supernatant was collected and filtered through a 0.22-μm glass fiber. Finally, each sample was measured using gas chromatography-mass spectrometry (GC-MS, Thermo Fisher Scientific, Waltham, MA, USA) and calibrated and evaluated using the internal standard method. To determine mPAEs, 1.0 ml of urine sample was mixed with 0.25 ml ammonium acetate buffer solution (pH = 6.5, Sigma) and 10 μl β-glucuronidase standard solution (85,000 U/ml, Sigma), and placed in a 37°C water bath for 2 h. Then, the sample mix solution was filtered by a 0.22-μm glass fiber filter for further analysis.

### 2.3. *mtDNA* methylation analyses

After a medical examination, fasting blood samples were collected from 39 individuals by venipuncture using vacuum anticoagulant tubes with 2 ml EDTA, followed by centrifugation at 3,000 rpm for 10 min to separate plasma. Then 39 samples were stored at −80°C until *mtDNA* methylation analysis for platelets.

First, 300 μl of plasma was removed from each sample, and platelet mitochondria were extracted using a DNA methylation kit (Zymo Research, Orange, CA, USA) and treated with bisulfite. A 24-μl system was prepared from the bisulfite-treated DNA, and the DNA was amplified by polymerase chain reaction (PCR). Then the DNA was amplified 45 times under the conditions of 5 min at 95°C, 15 s at 95°C, 30 s at 52°C, 15 s at 72°C, and 5 min at 72°C. The PCR amplifications were conducted in a PCR amplifier (BIO-RAD, USA). The treated samples were run on 0.8% agarose gel and visualized under UV light. Finally, the rate of *mtDNA* methylation was tested by the PyroMark Q48 autoprep pyrophosphate sequencer (QIAGEN, Hilden, Germany) (26).

### 2.4. Instrumental analysis

Phthalates were determined by Agilent 8890-5977B GC-MS. The gas chromatography (GC) conditions were as follows: column, DB-5MS UI (30 m × 0.25 mm × 0.25 μm); carrier gas, helium. The injection volume was 1 mul splitless, with an injection port temperature of 250°C. The carrier gas was helium, with a flow rate of 1.0 ml/min (constant flow mode). The temperature program was as follows: the initial temperature was 80°C for 60 s, followed by 300°C (10°C/min) for 120 s, and then 340°C (5°C/min) for 60 s. MS conditions were as follows: scan mode: SIM, electron energy: 70 eV; interface temperature: 280°C; ion source temperature: 230°C.

High-performance liquid chromatography-mass spectrometry (HPLC-MS; Agilent 1290, USA) was used to detect mPAEs. HPLC Nexera LC-30A high-performance liquid chromatograph (Shimadzu). The parameters of HPLC were: chromatographic column: Kinetex XB-C18-100A LC column (100 × 3 mm, 2.6 μm; Phenomenex, USA); flow rate: 0.3 ml/min; injection volume: 10 μl; column temperature: 40°C. Mobile phase A contained an aqueous solution with 0.1% formic acid, and mobile phase B contained methanol (HPLC grade; Fisher Scientific, Houston, TX, USA). The mobile phase gradient program is listed in Supplementary Table 1.

*mtDNA* methylation was determined using a pyrosequencer (PyroMark Q48 Autoprep, QIAGEN, Hilden, Germany). The process was as follows: (1) 200 μl of enzyme-free water was added to the instrument's reagent clip for cleaning before sequencing each time, and the cleaning step was repeated once; (2) dNTP, denaturation buffer, enzyme mixture, mixed substrate, and annealing buffer were added to the instrument; (3) After the instrument was detected, 3 μl of magnetic beads (PyroMark Q48 Magnetic Beads) and 10 μl of the sample to be tested were added. The 48-well sample tray was added to the instrument, and then it was put on the instrument for *mtDNA* methylation sequencing; (4) The reagent clips were cleaned again after the test. *mtDNA* sequences used for targeted bisulfite sequencing are listed in Supplementary Table 2.

### 2.5. Quality assurance and quality control

Blank analysis was performed on each batch of samples. The recoveries of all the target analytes in the matrix-spiked samples were 85–112% (PAEs) and 89–115% (mPAEs). The relative standard deviations were below 10%. No obvious ionization restraint or enhancement was discovered, and the matrix effects of the target analyte were on the scale of 86–110% (PAEs) and 85–106% (mPAEs). The limit of quantification (LOQ) and limit of detection (LOD) were defined as 10 and three times the signal-to-noise ratio (S/N), respectively. The LOQ and LOD were estimated at 0.02–1.27 ng/ml and 0.006–0.4 ng/ml for PAEs, and 0.01–6.54 ng/ml and 0.003–2.1 ng/ml for mPAEs. Concentrations below the LOQ were specified as zero values for statistical analysis.

Strict QA/QC procedures were also carried out during the analysis of *mtDNA* methylation. Every equipment was sterilized, non-pyrogenic, and DNAse/RNAse-free during the analysis of *mtDNA* methylation, and then two quality controls were included. In each 96-well plate, two blank samples were set as negative controls in PCR. Two samples with known global DNA methylation levels, namely, low (1%) and high (99%), were added as positive controls in pyrophosphate sequencing. Finally, two duplicate pyrosequencing analyses were performed for each DNA sequence (26).

### 2.6. Statistical analysis

This study used R 4.1.0 software from Origin 2018 and SPSS software for data analysis. In this study, the concentrations of analytes below LOD were set to zero. Considering that PAE exposure may affect *mtDNA* methylation levels and lipid levels, we analyzed the relationships between PAE exposure, *mtDNA* methylation, and lipids by adjusting a generalized linear model of several covariates (including age, BMI, and seniority). Different PAEs and mPAEs were included in the generalized linear model together with age, BMI, and seniority covariates as independent variables. Since the levels of triglycerides and some *mtDNA* did not correspond to the normal distribution, the natural logarithm was used for statistical analysis (Shapiro-test *P* < 0.05). In addition, generalized linear models were used to analyze the relationships between *mtDNA* methylation, *in vivo* pollutants, and lipid levels after adjusting for age, BMI, and seniority. The *p*-value of < 0.05 was considered to indicate statistical significance and the *p*-value of < 0.01 was considered to indicate high significance.

### 2.7. Health calculations

The EDI (μg/kg bw/day) of PAE exposure was evaluated by the mPAEs values in urinary samples using the following equation (1, 5, 36)**:**


(1) $$\begin{array}{l} EDI=\frac{UCm\times UV\times MWp}{Fue\times bw\times MWm}+UCp \end{array}$$

Where UC_m_ (ng/ml) is the concentration of urinary mPAE, UV (2.0 L/day) is the average adult volume of urine every day (37). Bw (kg) is the body weight of the participant. UC_p_ (ng/ml) is the urinary PAE concentration. The EDI of DEHP is the sum of the calculated DEHP concentrations based on DEHP, MEHP, MEHHP, MECPP, and MEOHP (11). MW_p_ is the molecular weight of PAE; MW_m_ (g/mol) is the molecular weight of mPAE; and F_ue_ is the molar fraction of mPAE. The values of MW_p_, MW_m_, and F_ue_ are listed in Supplementary Table 3.

The hazard quotient (HQ) for exposure to PAEs was computed using equation (38):


(2) $$\begin{array}{l} HQ=\frac{EDI}{TDIorRfD} \end{array}$$

EDI (μg/kg bw/day) is the estimated total daily intake of PAE; TDI (μg/kg bw/day) is recommended by the European Food Safety Authority (EFSA); the TDI values of DiBP and DEHP were 10 and 50 μg/kg bw/day, respectively. RfD (μg/kg bw/day) is recommended by the Environmental Protection Agency (EPA), the RfD values for DEP, DiBP, and DEHP were 800, 20, and 100 μg/kg bw/day. An HQ < 1 is considered safe, whereas an HQ > 1 indicates that the chemical may have potential adverse health effects on humans.

HI was estimated by summing the different values of HQ as follows:


(3) $$\begin{array}{l} \text{H}{\text{I}}_{TDI}=HQ_{D\text{i}BP_{TDI}}+H{Q_{DEHP}}_{{}_{TDI}} \end{array}$$

(4) $$\begin{array}{l} HI_{RfD}=HQ_{DEP_{RfD}}+HQ_{DiBP_{RfD}}+H{Q_{DEHP}}_{{}_{RfD}} \end{array}$$

## 3. Results

### 3.1. The level and relationship of PAEs and mPAEs

Out of 16 PAEs, nine were detected in urine samples in this study, including DMP, DEP, DiBP, DBP, BMPP, DPP, DEHP, DPHP, and DNOP. The detection rates ranged from 2.56 to 92.31%. The highest median concentration was DIBP (2.43 ng/ml), followed by DBP (2.40 ng/ml), DPP (0.55 ng/ml), DMP (0.51 ng/ml), and DPHP (0.06 ng/ml) as shown in Table 2.

**Table 2:** Urinary concentrations (ng/ml) of PAEs and mPAEs in urine from Tianjin.

**Class**	**Analyte**	**Detection** **Rates (%)**	**Analyte concentration (ng/ml)**			
			**Median**	**Mean**	**25th percentile**	**75th percentile**
PAE	DMP	64.10	0.51	3.44	–	1.53
	DEP	48.72	–	0.61	2.43	6.30
	DiBP	84.62	2.43	5.00	–	0.53
	DBP	89.74	2.40	7.09	0.94	6.45
	BMPP	92.31	–	1.26	0.43	0.66
	DPP	7.69	0.55	3.06	–	–
	DEHP	20.51	–	1.56	–	–
	DNOP	2.56	–	1.28	–	–
	DPHP	51.28	0.06	2.58	–	1.76
	ΣPAE		7.03	16.17	3.53	18.80
mPAE	MMP	30.77	–	1.44	–	0.27
	MEP	100	4.74	6.67	3.27	7.63
	MiBP	100	28.63	34.46	14.53	46.20
	MBP	100	28.87	37.68	15.14	43.21
	MEHP	100	1.63	5.82	1.03	4.24
	MBZP	82.05	0.06	0.51	0.01	0.16
	MOP	100	0.12	0.19	0.09	0.18
	MEHHP	100	4.00	7.32	1.88	7.17
	MECPP	100	7.23	16.34	4.77	15.34
	MEOHP	100	2.11	4.29	1.13	4.51
	ΣmPAE		89.43	115.63	52.32	141.82

“−” No value due to being below the limit of detection.

**Figure 1:**
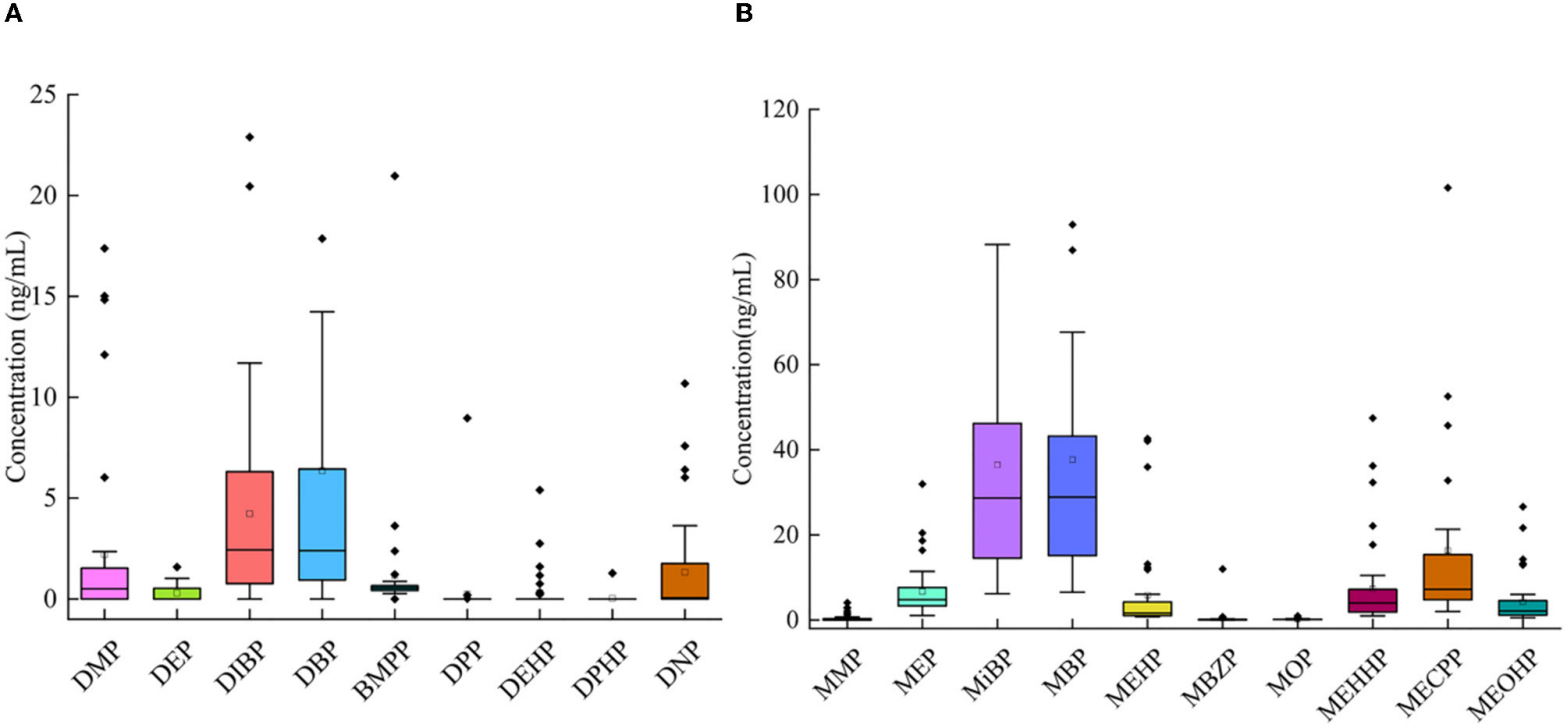
Concentrations of 9 PAEs **(A)** and 10 mPAEs **(B)** in urine of all participants from Tianjin.

**Figure 2:**
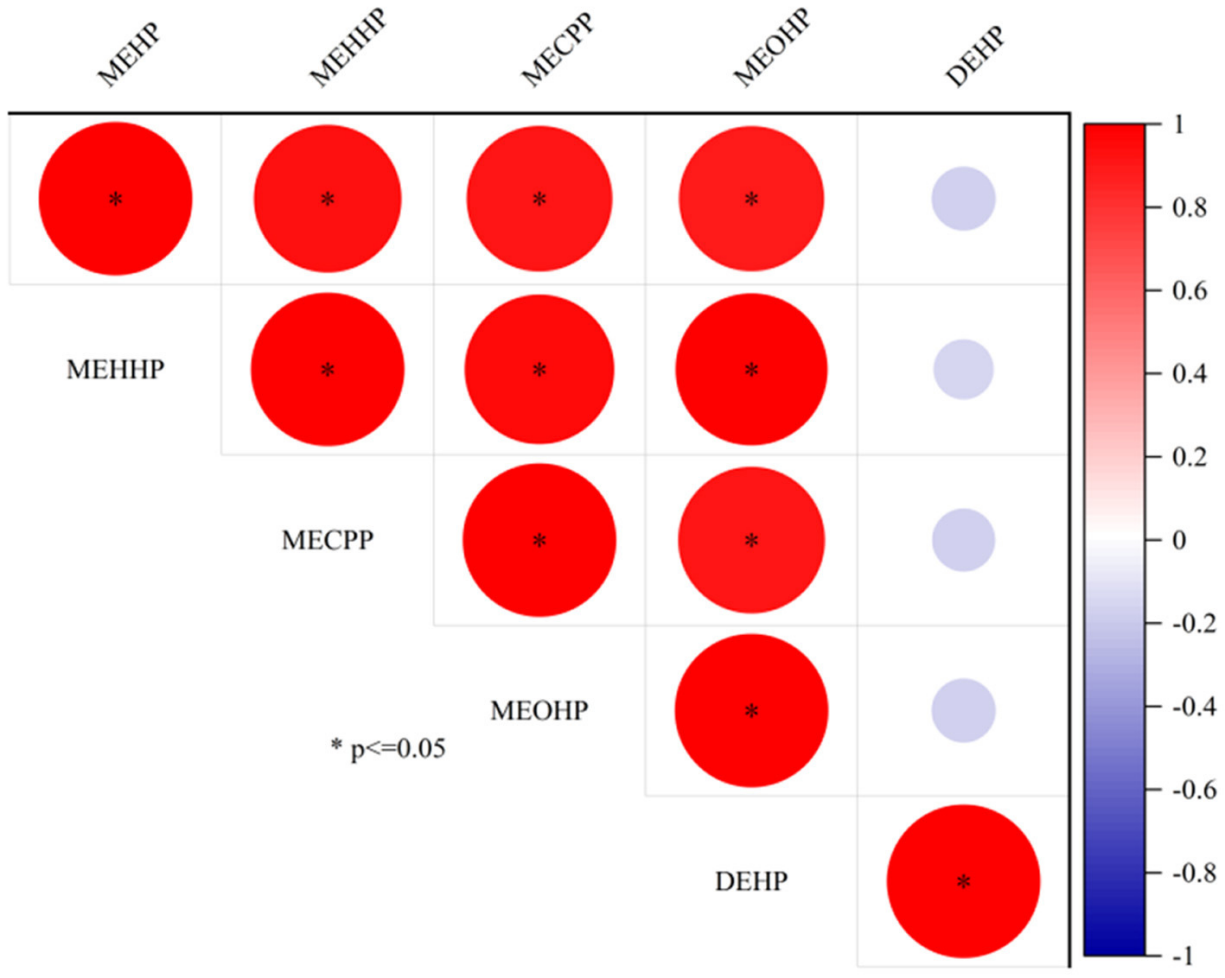
The correlation between DEHP and DEHP metabolite concentrations in urine.

In the samples, 10 mPAEs, including MMP, MEP, MiBP, MBP, MBZP, and MOP, and four metabolites of DEHP (including MEHP, MEHHP, MECPP, and MEOHP) were detected. MiBP, MECPP, MEHHP, MEP, MEHP, MEOHP, MBP, and MOP were found in 100% of the samples, whereas MMP and MBZP were less frequently detected (30.77 and 82.05%, respectively). The highest median concentration was MBP (28.87 ng/ml), followed by MiBP (28.63 ng/ml), MECPP (7.23 ng/ml), MEP (4.74 ng/ml), MEHHP (4.00 ng/ml), MEOHP (2.11 ng/ml), MEHP (1.63 ng/ml), MOP (0.12 ng/ml), and MBZP (0.06 ng/ml). Figures 1A, B show that higher concentrations were found for MiBP and MBP, which correspond to the concentrations of their parent diesters (DIBP and DBP). The average contributions of nine PAEs (Σ9 PAEs) and 10 mPAEs (Σ10 mPAEs) were 12.3 and 87.7%, respectively. The results showed a significant positive correlation between the urinary metabolites of DEHP (MEHP, MEHHP, MEOHP, and MECPP) (*p* < 0.05), respectively (Figure 2).

### 3.2. The relationship between urinary concentrations of PAEs and mPAEs with lipid levels

Using a generalized linear model, we tested associations between PAEs and mPAEs with lipid levels (including TC, triglycerides, HDL-C, and LDL-C). PAEs and lipid levels did not have a significant relationship (Supplementary Table 4). Both MEP and MBZP showed a positive influence on triglycerides (β = 0.90, *p* < 0.05; β *f*= 0.85, *p* < 0.001, respectively) (Table 3). Because a high level of triglycerides is one of the causes of CVDs (18), MEP and MBZP may increase the risk of CVDs.

**Table 3:** Associations between mPAEs concentrations with lipid levels.

**Variable**		**TC (mmol/ L)**	**LDL-C (mmol/ L)**	**HDL-C (mmol/ L)**	**Trigly cerides (mmol/ L)**
MMP	β	−0.07	0.02	−0.38	−0.12
	SD	0.12	0.14	0.28	0.28
	*p*	0.58	0.88	0.19	0.67
MEP	SD	−0.02	−0.24	−0.34	0.90
	SD	0.20	0.23	0.49	0.44
	*p*	0.93	0.30	0.49	0.048^*^
MiBP	SD	−0.48	−1.07	1.47	−0.62
	SD	0.44	0.48	1.04	1.02
	*p*	0.28	0.03^*^	0.17	0.55
MBP	β	−0.41	−1.01	1.75	−0.82
	SD	0.47	0.51	1.09	1.07
	*p*	0.38	0.06	0.12	0.45
MEHP	β	0.06	0.26	−0.45	−0.01
	SD	0.31	0.35	0.73	0.70
	*p*	0.84	0.47	0.55	0.99
MBZP	SD	0.02	−0.10	−0.19	0.85
	SD	0.12	0.13	0.28	0.22
	*p*	0.84	0.47	0.50	0.00^**^
MOP	SD	0.01	0.03	−0.02	−0.03
	SD	0.03	0.04	0.08	0.08
	*p*	0.83	0.49	0.78	0.72
MEHHP	SD	−0.18	−0.04	−0.88	0.08
	SD	0.29	0.34	0.69	0.67
	*p*	0.54	0.91	0.21	0.91
MECPP	SD	−0.13	0.11	−0.88	−0.02
	SD	0.45	0.52	1.07	1.03
	*p*	0.78	0.84	0.42	0.98
MEOHP	SD	−0.13	−0.10	−0.54	0.14
	SD	0.22	0.25	0.52	0.50
	*p*	0.56	0.70	0.30	0.79
ΣmPAE	?	−0.54	−1.03	1.08	−0.65
	SD	0.77	0.87	1.84	1.76
	*p*	0.48	0.24	0.56	0.72

^**^p < 0.01; ^*^p < 0.05.

### 3.3. The relationship between *mtDNA* methylation and lipid levels

For methylation analysis, six genes, including *MT-COX1, MT-COX2, MT-COX3, MT-ATP6, MT-ATP8*, and *MT-ND5*, were selected. Genetic mutations in cyclooxygenase (COX) genes are significantly associated with fatal metabolic disorders (39). ATPase subunit 6 (ATP6) and ATPase subunit 8 (ATP8) firsthand affect ATP synthesis and energy metabolism (40). NADH dehydrogenase subunit 5 (ND5) is the vital gene encoding NADH dehydrogenase. Hence, these genes mainly affect organs with high energy demands (41), such as the heart.

**Figure 3:**
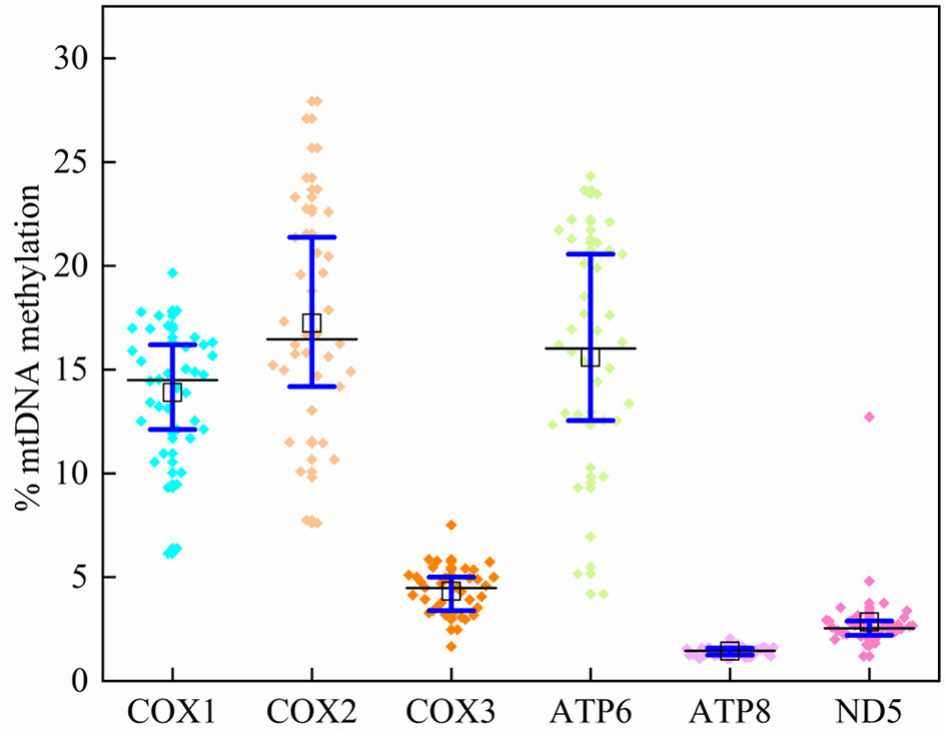
Mitochondrial DNA methylation levels at six genes.

In 39 plasma samples, the mean methylation level [interquartile range (IQR) 25th−75th percentile] in the *COX1* gene was 14.02 (12.23–16.17)%, 14.73 (11.12–17.35)% for *COX2*, 2.34 (2.12–2.62)% for *COX3*, 15.59 (12.55–20.45)% for *ATP6*, 1.43 (1.25–1.59)% for *ATP8*, and 2.84 (2.21–2.89)% for *ND5* (Supplementary Table 5; Figure 3). The average methylation rates of *MT-COX2* and *MT-ATP8* were positively correlated with triglycerides (*p* < 0.05, respectively), indicating that the *mtDNA* methylation of specific genes could induce CVDs, which needs to be further explored (Table 4).

**Table 4:** Associations between lipid levels and the *mtDNA* methylation levels.

**Variable**	***COX1***			***COX2***			***COX3***			***ATP6***			***ATP8***			***ND5***		
	β	**SD**	***p***	β	**SD**	***p***	β	**SD**	***p***	β	**SD**	***p***	β	**SD**	***p***	β	**SD**	***p***
TC (mmol/L)	0.32	0.62	0.61	1.22	0.85	0.16	0.03	0.08	0.71	0.40	1.12	0.72	0.05	0.05	0.32	−0.05	0.08	0.55
LDL-C (mmol/L)	−0.37	0.71	0.60	0.40	1.00	0.69	−0.06	0.09	0.56	−0.36	1.29	0.78	−0.03	0.05	0.62	−0.04	0.09	0.65
HDL-C (mmol/L)	−0.02	1.50	0.99	−0.73	2.09	0.73	−0.23	0.19	0.25	0.10	2.71	0.97	−0.02	0.12	0.84	0.03	0.18	0.87
Triglycerides (mmol/L)	2.59	1.36	0.07	3.94	1.88	0.04*	0.21	0.19	0.28	2.90	2.53	0.26	0.25	0.11	0.02*	−0.05	0.18	0.79

^**^p < 0.01; ^*^p < 0.05.

### 3.4. The relationships between PAEs and mPAEs concentrations with *mtDNA* methylation levels

Relationships of PAEs and mPAEs concentrations with *mtDNA* methylation levels were analyzed by generalized linear models. A 1-ng/ml increment in DEP concentration was associated with a 2.44 and 0.50% decrease in *MT-COX1* and *MT-COX3* methylation rates (*p* < 0.05, *p* = 0.001, respectively) among all participants (Table 5). Each 1 ng/ml increase in DBP and DiBP concentrations was associated with a 1.28 and 1.78% decrease in the *MT-ATP6* methylation rates (*p* < 0.05, respectively). The *MT-ND5* methylation rate was positively associated with BMPP (β = 0.41, *p* < 0.05), DPP (β = 0.66, *p* < 0.05), MiBP (β = 0.06, *p* < 0.05), and MBP (β = 0.06, *p* < 0.05). These results suggested that the *mtDNA* methylation level was susceptible to changes in response to PAE exposure.

**Table 5:** The associations between the *mtDNA* methylation levels with PAEs and mPAEs concentrations.

**Variable**	***COX1***			***COX2***			***COX3***			***ATP6***			***ATP8***			***ND5***		
	β	**SD**	***p*** **-value**	β	**SD**	***p*** **-value**	β	**SD**	***p*** **-value**	β	**SD**	***p*** **-value**	β	**SD**	***p*** **-value**	β	**SD**	***p*** **-value**
DMP	−0.11	0.43	0.80	0.32	0.60	0.60	−0.10	0.05	0.08	−1.53	0.74	0.04*	−0.03	0.03	0.46	0.04	0.05	0.46
DEP	−2.44	1.18	0.045*	−1.53	1.72	0.38	−0.50	0.14	0.001**	−1.92	2.23	0.39	−0.14	0.09	0.15	0.13	0.15	0.41
DIBP	−0.56	0.40	0.17	−0.15	0.58	0.80	−0.11	0.05	0.04*	−1.78	0.69	0.01*	−0.03	0.03	0.42	0.03	0.05	0.56
DBP	−0.06	0.30	0.83	0.03	0.41	0.94	−0.09	0.04	0.02*	−1.28	0.49	0.01*	−0.04	0.02	0.11	0.03	0.04	0.50
BMPP	−1.22	0.69	0.09	−1.10	0.98	0.27	0.03	0.09	0.78	−1.14	1.28	0.38	−0.08	0.05	0.13	0.41	0.06	0.00**
DPP	−1.44	1.01	0.16	−1.40	1.42	0.33	0.12	0.14	0.38	0.18	1.87	0.92	−0.05	0.08	0.56	0.66	0.06	0.00**
DEHP	0.21	0.95	0.83	1.34	1.31	0.31	−0.08	0.13	0.54	−1.08	1.71	0.53	0.01	0.07	0.88	−0.07	0.12	0.56
DNOP	−1.34	2.74	0.63	−3.61	3.80	0.35	0.04	0.36	0.91	−9.16	4.74	0.06	−0.20	0.21	0.35	0.12	0.34	0.72
DPHP	0.45	0.54	0.42	0.84	0.75	0.27	−0.05	0.07	0.49	−1.67	0.95	0.09	−0.01	0.04	0.90	−0.03	0.07	0.62
ΣPAE	−0.15	0.23	0.52	0.03	0.32	0.93	−0.06	0.03	0.06	−1.11	0.37	0.004**	−0.03	0.02	0.13	0.04	0.03	0.11
MMP	−0.90	0.85	0.29	−1.46	1.17	0.22	0.04	0.11	0.74	2.25	1.51	0.14	0.05	0.07	0.44	0.14	0.10	0.20
MEP	0.36	0.51	0.49	1.10	0.69	0.12	0.06	0.07	0.39	0.17	0.92	0.85	0.05	0.05	0.30	0.09	0.06	0.14
MiBP	−0.03	0.23	0.90	0.15	0.32	0.65	0.01	0.03	0.76	−0.14	0.42	0.75	0.00	0.02	0.84	0.06	0.03	0.04*
MBP	−0.01	0.22	0.95	0.15	0.31	0.63	0.01	0.03	0.70	−0.11	0.40	0.78	0.00	0.02	0.81	0.06	0.03	0.04*
MEHP	0.26	0.34	0.44	0.46	0.47	0.33	0.01	0.04	0.87	0.58	0.60	0.34	0.02	0.03	0.43	0.00	0.04	0.94
MBZP	1.16	0.88	0.19	0.75	1.25	0.55	0.05	0.12	0.67	2.28	1.58	0.16	0.02	0.07	0.75	0.09	0.11	0.43
MOP	0.44	3.02	0.88	2.16	4.20	0.61	−0.04	0.40	0.92	7.54	5.31	0.16	0.19	0.23	0.42	−0.05	0.37	0.89
MEHHP	0.33	0.35	0.36	0.64	0.48	0.19	0.03	0.05	0.48	0.34	0.64	0.59	0.00	0.03	0.87	0.01	0.04	0.78
MECPP	0.20	0.23	0.40	0.38	0.32	0.24	0.02	0.03	0.56	0.27	0.41	0.52	0.01	0.02	0.55	0.01	0.03	0.83
MEOHP	0.54	0.46	0.26	1.05	0.64	0.11	0.05	0.06	0.44	0.75	0.84	0.38	0.01	0.04	0.69	0.03	0.06	0.60
ΣmPAE	0.05	0.13	0.70	0.17	0.19	0.38	0.01	0.02	0.54	0.07	0.24	0.79	0.00	0.01	0.70	0.03	0.02	0.10

^**^p < 0.01; ^*^p < 0.05.

### 3.5. Mediation analysis on *mtDNA* methylation and lipid levels

The triglyceride levels were significantly associated with MEP concentration and *MT-ATP8* methylation rate (*p* < 0.05, β = 0.90; *p* = 0.02, β = 0.25, respectively). To clarify the role of MEPs in the epigenetic regulation of the mitochondrial genome, we conducted a mediation analysis to examine the MT-ATP8 methylation rate with MEP and triglyceride levels (Table 6). However, we did not find the mediated effect of the above exposure levels on triglycerides through the *mtDNA* methylation pathway (*p* > 0.05).

**Table 6:** The mediation effects of MT-ATP8 methylation on the association of MEP levels with triglycerides.

**Model**		**Unstandardized coefficients**		**Standardized coefficients**	**T**	**Significance**
		**B**	**Standard error**	**Beta**		
1	Constant	1.254	0.246		5.097	0.000
	MEP	0.065	0.027	0.371	2.396	0.022
2	Constant	0.265	0.747		0.355	0.725
	MEP	0.037	0.033	0.214	1.132	0.265
	*ATP8*	0.788	0.564	0.265	1.398	0.171

Dependent variable: triglycerides. “Beta” is the coefficient of the independent variable (exposure levels) when regressing the mediator (MT-ATP8 methylation levels) on the independent variable.

### 3.6. Human risk assessment

The EDIs, HQs, and HIs were calculated based on the levels of PAEs (DEP, DiBP, and DEHP). The median EDIs of DEP, DiBP, and DEHP were 0.438, 3.676, and 4.374 μg/kg bw/day, with details listed in Table 7. HQ showed a risk for a single PAE, while HI indicated a cumulative risk for several PAEs (calculated by the sum of DEP, DiBP, and DEHP). HQ<1 and HI<1 are considered significant daily intake doses and safe adverse effects caused by PAEs. In this study, 10% of participants showed HI_RfD_>1. Additionally, it should be noted that 12 of the 39 men in this study showed HI_TDI_>1, indicating that ~31% of the population has potential health risks.

**Table 7:** Estimated daily intake (μg/kg bw/day), hazard quotient, and hazard index for 3 PAEs in 39 men.

**Analyte**	**EDI**			**HQ**_**RfD**_			**HI**	**HQ**_**TDI**_			**HI**
	**Median**	**95th**	**Max**	**Median**	**95th**	**Max**	***N*** >**1**	**Median**	**95th**	**Max**	***N*** >**1**
DEP	0.44	1.65	1.75	0.001	0.002	0.002	-	-	-	-	-
DIBP	3.68	21.77	23.44	0.18	1.09	1.17	3	0.37	2.18	2.34	7
DEHP	4.37	56.75	59.52	0.04	0.57	0.60	-	0.09	1.14	1.19	3
HI (%)	-	-	-	0.28	1.28	1.38	10.26	0.56	2.55	2.69	31

EDI, estimated daily intake; HI, hazard index; TDI, tolerable daily intake; RfD, the reference doses.

## 4. Discussion

In this study, 9 PAEs and 10 mPAEs were detected in the urine of 39 men in Tianjin, with the average detection rate exceeding 30.77%. The detection rate in this study was similar to that of 88 male volunteers in Tianjin, China (31.8%) (42), but lower than that of 84 primiparas in Shenzhen, China (73%) (8) and 84 male adults in Fujian, China (64.3%) (43). In this study, the most widely detected were MBP, MiBP, and MEHP (100%, respectively), higher than the detection frequency of the general population in Guangzhou, China (84.2, 46.3, and 60.4%, respectively) (44), and higher than the prospective cohort of 2,298 children in Fujian, China (99.76, 97.23, and 54.52%, respectively) (45). This finding could be explained by the following factors: (1) PAE exposure characteristics could vary widely in different geographical areas. (2) Some factors, such as individual genetic susceptibility, could influence pollutant metabolism.

The primary metabolites of DEHP were MEHP, and the secondary metabolites were MECPP, MEHHP, and MEOHP. Strongly positive relationships were found between MEHP, MEHHP, MEOHP, MECPP, and DEHP (Figure 2), suggesting that these metabolites originated from the same exposure sources. We found that the median concentration of the three secondary metabolites of DEHP was 1.29–4.43 times higher than that of the primary metabolites, a result similar to that found in Canadian men (2.38–4.47 times) (46), which could be explained by the short half-life time of the MEHP.

Except for DEHP, the metabolism of other components is dominated by primary metabolism. MBP and MiBP were the most abundant compounds, with a median concentration of 28.87 and 28.63 ng/ml, respectively. The possible reason might be that their parent compounds (DBP and DiBP) are commonly used plasticizers. Compared to the results reported in the literature (Table 8), the concentrations of MBP in this study were much lower than that of primiparas in Shenzhen, China (median: 139 ng/ml), the general population in Guangzhou, China (median: 89.4 ng/ml), children in Fujian, China (median: 172.6 ng/ml), and the general population in Fujian, China (median: 75.3 ng/ml), but higher than that of pregnant women in Massachusetts, USA (median: 16.5 ng/ml) and men in Canada (median: 16.7 ng/ml) (8, 43–47). In addition, the median concentration of MiBP was close to that reported for children in Fujian, China (median: 23.16 ng/ml) (45). They were generally higher than those pregnant women in Massachusetts, USA (median: 7.57 ng/ml) and men in Canada (median: 12.6 ng/ml), but much lower than those men in Tianjin, China (median: 190 ng/ml) (42, 46, 47). This finding could be explained by the following factors: (1) Different countries have different levels of PAEs in the industry; and (2) different climate scenarios have different effects on the exposure levels (48).

**Table 8:** Comparison of concentrations and levels of mPAEs in this study and previous studies (ng/ml).

**Study**		**MMP**	**MEP**	**MiBP**	**MBP**	**MEHP**	**MBZP**	**MOP**	**MEHHP**	**MECPP**	**MEOHP**
This study	Median	0	4.74	28.63	28.87	1.63	0.06	0.12	4.00	7.23	2.11
	Mean	1.44	6.67	34.46	37.68	5.82	0.51	0.19	7.32	16.34	4.29
	Detection rate	30.77	100	100	100	100	82.5	100	100	100	100
Shenzhen, China (8)	Median	4.59	4.87		139	2.86	0.24	-	5.45		4.31
	Mean	5.21	14.4		174	3.74	0.36	-	9.38		7.73
	Detection rate	97	98		100	100	73	0	100		100
Massachusetts, USA (47)	Median		121	7.57	16.5	9.07	6.38		27.5	34.9	15.3
Guangzhou, China (44)	Median	14.3	5.26	< LOD	89.4	3.21	< LOD		4.61		6.82
	Mean	17.4	20.2	50.4	137	10.90	4.42		11.3		14.9
	Detection rate	64.2	76.3	46.3	84.2	60.40	29.6		67.9		75
Fujian, China (45)	Median	32.79	8.071	23.16	172.60	8.10			17.72		3.52
	Detection rate	100	99.4	97.23	99.76	54.52			99.88		84.44
Fujian, China (43)	Median	36.40	36.70		75.30	3.20	0.18		7.40	13.64	3.76
	Mean	81.26	66.22		105.65	4.22	1.70		10.52	18.53	4.72
	Detection rate	100	100		100	100	64.3		100	100	100
Canada (46)	Median	2.1	48.1	12.6	16.7	3.2	4.9	0.4	11.4	14.3	7.6
	Mean	3.5	191.4	29.7	27.4	6.6	10.0	0.5	22.9	31.1	15.0
	Detection rate	88.7	100	100	99.3	98	98.7	7.3	100	100	100
Tianjin, China (42)	Median	4.38	8.81	190		12.9	4.10		1.85	22.3	
	Mean	7.04	18.8	192		14.1	4.64		4.06	29.6	0.94
	Detection rate	100	100	100		100	100		100	100	31.8

In this study, the TDI value of DiBP exceeded 17.9% of men, and the TDI of DEHP exceeded 7.6%. For 39 men, 10 and 31% of HI values exceeded the RfD and TDI thresholds, respectively. Compared to our study, Chen et al. (8) found that 2 and 5% of EDI values in primiparas were greater than TDI and RfD values, and ~55% of HI_TDI_ values exceeded 1 in Shenzhen, China. Gao et al. (11) found that 40% of young adults in China showed HI values exceeding 1. These results demonstrated that adults probably have a health risk from PAE exposure in China. Given that PAE exposure has been associated with substantial adverse health effects, more attention should be paid to the cumulative risks of PAE exposure in Chinese adults.

This study speculates that mitochondrial epigenetic alterations can link PAE to adverse cardiovascular outcomes. We discovered that MEP and MBZP showed a significant relationship with triglycerides. Recently, most investigations have explored the PAEs associated with CVD risk (12, 14). PAE exposure levels have been related to oxidative stress, possibly through the activation of peroxisome proliferator-activated receptors or changes in mitochondrial membrane potential and permeability (49). Rosado-Berrios et al. (50) found that MEHP and DEHP affected mitochondrial membrane potential and promoted reactive oxygen species generation, then proved oxidative stress is a core underlying mechanism in the progression of CVDs (51). A meta-study showed that PAEs play a role similar to estrogen in the human body, which may bind to estrogen receptor α or receptors α and γ (pPARα and pPARγ) and damage β cell function to control carbohydrate metabolism and fat production (19). Given that triglycerides decompose into carbohydrates and water after liver metabolism, it is reasonable to assume that PAEs affect triglyceride metabolism, thereby leading to metabolic disorders and triglyceride accumulation (52, 53). As an essential subunit in ATP synthase, ATP8 directly affects ATP synthesis and energy metabolism (26). Therefore, the ATP8 gene plays a vital role in triglyceride metabolism. PAEs, *mtDNA* methylation, and triglyceride levels may have a potential association.

Through the statistical analysis, no mediator effect of PAE exposure on triglycerides through *MT-ATP8* expression was observed. However, it is still worth noting that we found that 10.26–31.00% HI exceeded 1 and had a broad range of *mtDNA* methylation levels (1.09–24.31%, more than 22-fold differences), which is potentially risky. Baccarelli et al. found that CVDs are associated with the methylation of different segments of *mtDNA* (19, 54, 55). Liu et al. (20) carried out a study with 110 participants, which found that due to air pollutants, methylation levels in the *MT-COX1, MT-ATP6*, and *MT-ATP8* regions were significantly lower, while *COX2* and *COX3* regions were obviously higher, providing a direction on the epigenetic regulation research of mitochondrial function.

This study was the first to relate urine PAE exposure and *mtDNA* methylation with lipid levels in men. PAE concentrations and health risks were assessed. In addition, the study evaluated the relationships between PAE levels and *mtDNA* methylation with lipid levels. The results provide a new direction to study PAEs and CVDs. However, some aspects could still be improved in this study. The small sample size in this study may only represent some of the population in Tianjin. The HI was only calculated with three PAEs due to the lack of parameters for other highly detectable PAEs. The relationships between PAEs, mPAEs concentrations, and *mtDNA* methylation levels with lipid levels might be influenced by other pollutants. Further studies are needed to explain the specific response relationship and the mechanisms of action on CVDs.

## 5. Conclusion

The risk assessments of the concentration accumulation of PAEs in men were analyzed in this study. MBP and MiBP were the dominant compounds in Chinese young men. We detected *mtDNA* methylation levels by using sequencing technology and assessed the associations between lipid levels with *mtDNA* methylation rates and PAEs. Significant associations between urine MEP concentrations and *MT-ATP8* methylation with triglycerides were found. Those findings supported that PAE exposure is a risk factor for CVDs. We also explored the mediating effects of PAEs on the association between *mtDNA* methylation and lipid levels of CVDs, but the mediating effect failed to be found.

## Data availability statement

The original contributions presented in the study are included in the article/Supplementary material, further inquiries can be directed to the corresponding authors.

## Ethics statement

The studies involving human participants were reviewed and approved by the Ethics Committee of Tianjin Medical University. The patients/participants provided their written informed consent to participate in this study. Written informed consent was obtained from the individual(s) for the publication of any potentially identifiable images or data included in this article.

## Author contributions

WL, JF, LZ, SS, TF, LG, CL, PL, and LW defined the research theme. WL and JF analyzed the data, and WL wrote the study. All authors have contributed to, read, and approved the manuscript.
