# Supplementary material for: Phthalates and phthalate metabolites in urine from Tianjin and implications for platelet mitochondrial DNA methylation
Source: Front Public Health. 2023 Apr 26;11:1108555. doi: 10.3389/fpubh.2023.1108555 (PMC10169620; doi:10.3389/fpubh.2023.1108555)
Supplement: Supplementary file 1 [file Data_Sheet_1.docx]

Supplementary Material

# Supplementary Tables

Table A.1 The mobile phase gradient program of mPAEs.

| Time（min） | A% | B% |
| --- | --- | --- |
| 0 | 90 | 10 |
| 0.5 | 90 | 10 |
| 6 | 50 | 50 |
| 7 | 10 | 90 |
| 8 | 10 | 90 |
| 10 | 90 | 10 |

Table A.2 MT-DNA primer sequences used for targeted bisulfite sequencing.

| Gene | primer | Sequence |
| --- | --- | --- |
| *MT-COX1* | Forward primer (5' to 3') | TATTAATTGGTTTTTTAGGGTTTAT |
|  | Reverse biotin primer (5' to 3') | CAACAAATCATTTCATATTACTTCC |
|  | Sequencing primer (5' to 3') | TATTTATAGTAGGAAT |
| *MT-COX2* | Forward primer (5' to 3') | TTTATGAGTTGTTTTTATATTAGGTTTAAA |
|  | Reverse biotin primer (5' to 3') | ACTCCACAAATTTCAAAACATTAAC |
|  | Sequencing primer (5' to 3') | TAAAAATAGATGTAAT |
| *MT-COX3* | Forward primer (5' to 3') | TATATTATTTGTTTAAAAAGGTTTT |
|  | Reverse biotin primer (5' to 3') | AATAAAAAACTCAAAAAAATCCTAC |
|  | Sequencing primer (5' to 3') | TATATTATTTGTTTAAAAAGGTTTT |
| *MT-ATP6* | Forward primer (5' to 3') | TTATAAATTTAGTTATGGTTATTTTTTTAT |
|  | Reverse biotin primer (5' to 3') | AAACTAAAACATTTTTAATCTTAAAAC |
|  | Sequencing primer (5' to 3') | TTATAAATTTAGTTATGGTTATTTTTTTAT |
| *MT-ATP8* | Forward primer (5' to 3') | AAATTATAATAAATTTTGAGAATTAAAATG |
|  | Reverse biotin primer (5' to 3') | AATAAACCTAAAATTATAAAAACAATAAAT |
|  | Sequencing primer (5' to 3') | AAATTATAATAAATTTTGAGAATTAAAATG |
| *MT-ND5* | Forward primer (5' to 3') | GTGATATATAAATTTAGATTTAAATATTAA |
|  | Reverse biotin primer (5' to 3') | TAAACAAAAAAAATATAATTCCTAC |
|  | Sequencing primer (5' to 3') | TTAATTTTAGTTAT |

Table A.3 The values of MW_p_, MW_m_ and F_ue_

| PAEs | MW_p_ (g/mol) | mPAEs | MW_m_ (g/mol) | F_ue_ |
| --- | --- | --- | --- | --- |
| DMP | 194.19 | MMP | 180.16 | 0.70 |
| DEP | 222.24 | MEP | 194.18 | 0.69 |
| DBP | 278.34 | MBP | 222.24 | 0.70 |
| DiBP | 278.34 | MiBP | 222.24 | 0.70 |
| DEHP | 390.56 | MEHP | 278.34 | 0.059 |
|  |  | MEOHP | 291.32 | 0.15 |
|  |  | MECPP | 308.33 | 0.185 |
|  |  | MEHHP | 293.33 | 0.23 |

MW_p_ is the molecular weight of PAEs; MW_m_ (g/mol) is the molecular weight of mPAEs; F_ue_ is the molar fraction of mPAEs

Table A.4 Associations between PAEs concentrations with lipid levels.

| Variable | | TC(mmol/L) | LDL-C(mmol/L) | HDL-C(mmol/L) | triglycerid（mmol） |
| --- | --- | --- | --- | --- | --- |
| DMP | β | -0.21 | 0.09 | -0.29 | -0.52 |
|  | SD | 0.24 | 0.28 | 0.58 | 0.54 |
|  | P | 0.38 | 0.76 | 0.62 | 0.34 |
| DEP | β | -0.05 | 0.02 | 0.03 | -0.23 |
|  | SD | 0.08 | 0.10 | 0.20 | 0.19 |
|  | P | 0.52 | 0.80 | 0.87 | 0.23 |
| DiBP | β | -0.15 | -0.07 | 0.43 | -0.77 |
|  | SD | 0.25 | 0.29 | 0.60 | 0.56 |
|  | P | 0.56 | 0.81 | 0.47 | 0.18 |
| DBP | β | -0.24 | 0.06 | -0.34 | -0.72 |
|  | SD | 0.35 | 0.40 | 0.84 | 0.79 |
|  | P | 0.49 | 0.87 | 0.68 | 0.37 |
| BMPP | β | -0.03 | 0.09 | -0.12 | -0.25 |
|  | SD | 0.15 | 0.17 | 0.35 | 0.33 |
|  | P | 0.85 | 0.58 | 0.74 | 0.45 |
| DPP | β | -0.08 | -0.06 | 0.03 | -0.17 |
|  | SD | 0.10 | 0.11 | 0.24 | 0.23 |
|  | P | 0.44 | 0.61 | 0.90 | 0.46 |
| DEHP | β | 0.05 | 0.07 | -0.07 | -0.02 |
|  | SD | 0.11 | 0.12 | 0.26 | 0.25 |
|  | P | 0.66 | 0.56 | 0.78 | 0.94 |
| DNOP | β | -0.05 | -0.05 | 0.00 | -0.05 |
|  | SD | 0.04 | 0.04 | 0.09 | 0.09 |
|  | P | 0.21 | 0.26 | 0.97 | 0.56 |
| DPHP | β | 0.26 | 0.34 | 0.14 | 0.22 |
|  | SD | 0.18 | 0.21 | 0.46 | 0.43 |
|  | P | 0.17 | 0.12 | 0.75 | 0.62 |
| ΣPAE | β | -0.29 | 0.15 | -0.02 | -1.24 |
|  | SD | 0.46 | 0.52 | 1.10 | 1.03 |
|  | P | 0.53 | 0.77 | 0.99 | 0.24 |

**p < 0.01; *p<0.05

Table A.5 The average methylation rate (interquartile range 25th-75th percentile IQR)

| *MT-DNA* | P5 | P25 | P50 | P75 | P95 | Mean ± SD |
| --- | --- | --- | --- | --- | --- | --- |
| *COX1* | 8.88 | 12.23 | 14.54 | 16.17 | 17.79 | 14.02±3.03 |
| *COX2* | 7.12 | 11.12 | 15.49 | 17.36 | 21.10 | 14.73±4.24 |
| *COX3* | 1.70 | 2.12 | 2.36 | 2.62 | 2.93 | 2.34±0.40 |
| *ATP6* | 5.44 | 12.55 | 16.02 | 20.45 | 23.49 | 15.59±5.48 |
| *ATP8* | 1.11 | 1.25 | 1.46 | 1.59 | 1.80 | 1.43±0.23 |
| *ND5* | 1.74 | 2.21 | 2.54 | 2.89 | 3.91 | 2.84±1.77 |
